# Encapsulation of MnS Nanocrystals into N, S-Co-doped Carbon as Anode Material for Full Cell Sodium-Ion Capacitors

**DOI:** 10.1007/s40820-020-0367-9

**Published:** 2020-01-22

**Authors:** Shaohui Li, Jingwei Chen, Jiaqing Xiong, Xuefei Gong, Jinghao Ciou, Pooi See Lee

**Affiliations:** 1 School of Materials Science and Engineering, Nanyang Technological University, Singapore, 639798 Singapore; 2 Singapore-HUJ Alliance for Research and Enterprise (SHARE), Nanomaterials for Energy and Water Nexus (NEW), Campus for Research Excellence and Technological Enterprise (CREATE), 1 Create way, Singapore, 138602 Singapore

**Keywords:** Sodium-ion capacitor, Nanocrystal, Co-doped carbon, Pseudocapacitive control behavior

## Abstract

Sodium-ion capacitors (SICs) have received increasing interest for grid stationary energy storage application due to their affordability, high power, and energy densities. The major challenge for SICs is to overcome the kinetics imbalance between faradaic anode and non-faradaic cathode. To boost the Na^+^ reaction kinetics, the present work demonstrated a high-rate MnS-based anode by embedding the MnS nanocrystals into the N, S-co-doped carbon matrix (MnS@NSC). Benefiting from the fast pseudocapacitive Na^+^ storage behavior, the resulting composite exhibits extraordinary rate capability (205.6 mAh g^−1^ at 10 A g^−1^) and outstanding cycling stability without notable degradation after 2000 cycles. A prototype SIC was demonstrated using MnS@NSC anode and N-doped porous carbon (NC) cathode; the obtained hybrid SIC device can display a high energy density of 139.8 Wh kg^−1^ and high power density of 11,500 W kg^−1^, as well as excellent cyclability with 84.5% capacitance retention after 3000 cycles. The superior electrochemical performance is contributed to downsizing of MnS and encapsulation of conductive N, S-co-doped carbon matrix, which not only promote the Na^+^ and electrons transport, but also buffer the volume variations and maintain the structure integrity during Na^+^ insertion/extraction, enabling its comparable fast reaction kinetics and cyclability with NC cathode.

**Electronic supplementary material:**

The online version of this article (10.1007/s40820-020-0367-9) contains supplementary material, which is available to authorized users.

## Highlights

- Downsizing of MnS and encapsulating by conductive N, S-co-doped carbon matrix (MnS@NSC) provide excellent reversible capacity, rate capability, and cycling stability in sodium-based electrolyte.
- The charge storage mechanism of MnS@NSC was analyzed, showing pseudocapacitive control behavior.
- The as-fabricated sodium-ion capacitor delivers excellent electrochemical performance.

**Electronic supplementary material:**

The online version of this article (10.1007/s40820-020-0367-9) contains supplementary material, which is available to authorized users.

## Graphical abstract

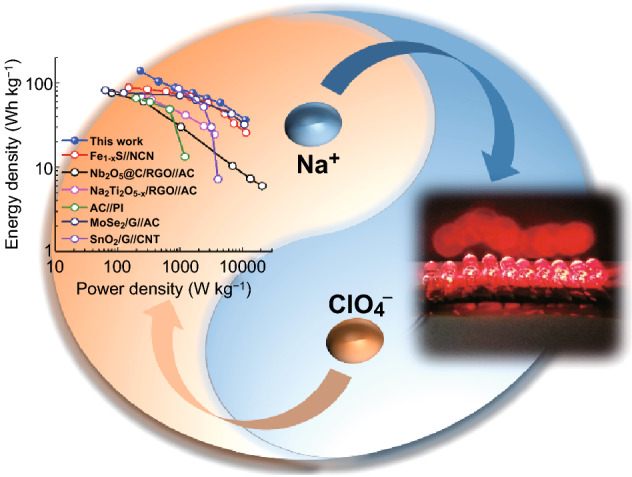

## Introduction

Electrochemical energy storage system is attracting extensive research interest due to the increasing demands of electric/hybrid vehicle, portable electronic devices, and scalable grid storage [1–4]. Currently, lithium-ion batteries (LIBs) have dominated the area of consumer electronics, sustainable automotive, and back-up power supply because of their high specific energy, high efficiency, and reliability [3, 5–7]. LIBs usually suffer from low power density and poor cycle life, furthermore, the growing cost of lithium source limited its sustainable development in large-scale utilization. Thus, it is important and urgent to explore an alternative energy storage system. Recently, research interests on sodium-ion batteries (SIBs) have rapidly grown not only due to the earth-abundant and evenly distributed sodium resource, but also due to its similar electrochemical behavior to lithium [8–12]. Unfortunately, similar to LIBs, SIBs are also limited by poor power density and relatively inferior cycling stability. To overcome these limitations, the concept of sodium-ion capacitor (SIC) was introduced in 2012 [3, 13]. SICs have the same configuration as lithium-ion capacitors (LICs), consisting of a faradaic battery anode and capacitor cathode, and thus deliver high power density without sacrificing the energy density [6, 14–17]. However, the slow diffusion-dominant reaction mechanisms along with the large volume changes due to the larger sodium ionic radii (0.98 Å) limits the rate capability and cycle life of SICs [2, 11]. It is challenging to develop low-cost and high-rate SIC anode materials with superior cycling stability to match with the fast capacitive sorption cathode.

To address this challenge, tremendous efforts are dedicated to investigate advanced anode materials for SIBs, including carbon-based materials [18–20], metal oxides [21–24], metal sulfides [8, 10, 25–28], metal selenides [29, 30], metal phosphides [31, 32], alloys [11, 33], covalent organic frameworks (COFs) [34], etc. Metal sulfides as promising SIB anode materials have merits of high theoretical capacities, better reversibility, and relatively higher electronic conductivity than metal oxides counterparts [26, 35–37]. Metal sulfides normally exhibit multielectron reaction mechanisms with sodium; the charging/discharging process combines both the intercalation and conversion reactions [10, 37]. Thus, severe volume changes are expected for metal sulfide electrodes, leading to poor rate capability and cycling stability. To overcome these drawbacks, typical methods such as downsizing, nanostructuring, and modification or their combinations are investigated to overcome the sluggish solid-state ion diffusion and buffer the issues caused by volume changes [8, 9, 30]. Among them, incorporating metal sulfides into the doped carbon matrix is an effective way to buffer the volume changes during charging/discharging process. Additionally, doped carbon materials with superior electrical conductivity can facilitate electron conduction in nanocomposite materials, shorten the ion diffusion pathway due to the reduced particle size, and inhibit the dissolution of polysulfides formed, thus realizing improved cycling stability with high energy or power densities [35, 36, 38]. Recently, MnS has attracted attention as a SIB anode candidate due to its natural abundance, environment friendliness, cost-effectiveness, and the theoretical capacity up to 616 mAh g^−1^ according to the electromotive force of 1.049 V [*E*^o^(MnS) vs. Na^+^/Na = 1.049 V] and Gibbs free energy change of − 202.50 kJ mol^−1^ [35, 39, 40]. Despite these advantages, MnS is still suffering from intrinsically poor electrical conductivity, sluggish electrochemical reactions with poor rate performance, drastic volume changes during cycling, dissolution/loss of polysulfides intermediates in electrolyte, and the agglomeration of nanoparticles in the charge/discharge processes, which seriously impede the use of MnS as high-rate anode for SICs [35, 38, 41, 42]. Although previous works have shown that embedding MnS nanostructures into carbonaceous materials can improve the electrochemical performance for sodium storage, most of the previously reported MnS-based anode materials suffer from agglomeration and structural collapses upon cycling, resulting in limited rate performance and cycling stability. According to the previous studies on metal sulfide materials, downsizing and incorporating metal sulfides into the carbon matrix are an effective way to achieve fast transport into the electrode and buffer the volume changes during charging/discharging process [31, 37]. However, so far, encapsulating MnS nanocrystals into carbon matrix to achieve fast electron/Na^+^ transport together with improved structural integrity for SICs still remains a challenge.

To mitigate these challenges, high-rate MnS-based SIB anode materials were demonstrated in this work by embedding MnS nanocrystals into the N, S-co-doped carbon matrix (MnS@NSC) using polyacrylonitrile (PAN) as both carbon and nitrogen source. The fully encapsulated MnS nanocrystals into the porous and conductive N, S-co-doped carbon matrix not only promote the facile access of Na^+^ and electrons, but also buffer the accumulated strain resulting from volume variations during Na^+^ insertion/extraction and maintain the structural integrity, thus displaying high reversibility and rate capability. Owing to these advantages, the MnS@NSC electrode exhibits excellent rate performance (464.3 mAh g^−1^ at 0.05 A g^−1^ and maintains 205.6 mAh g^−1^ at 10 A g^−1^) and outstanding cycling stability without notable degradation after 2000 cycles. By coupling the anode with a N-doped carbon (NC) cathode, the assembled SIC full cell can deliver energy density as high as 139.8 Wh kg^−1^ and power density of 11,500 W kg^−1^. The capacity retention achieved is 84.5% after 3000 cycles, demonstrating the superiority of MnS@NSC as promising anode candidate for SIC.

## Experimental

### Materials Preparation

#### Preparation of Mn-PAN Complex

0.4 g of polyacrylonitrile (PAN) and 1.376 g of Mn(CH_3_COO)_2_·4H_2_O were dissolved into 10 mL *N*,*N*-dimethylformamide (DMF) under magnetic stirring for 2 h at 60 °C. Then, the as-prepared solution was added dropwise to a mixed solution of ethylene glycol (EG) and ethanol (v:v = 5:2) under vigorous stirring. After finishing the infusion, the resultant mixture was stirred at 180 °C and refluxed for 9 h. When the temperature cooled down, the product of Mn-PAN complex was collected by filtration, followed by drying at 80 °C in the vacuum oven overnight.

#### Preparation of MnS@NSC Anode Composite

The as-obtained Mn-PAN complex was first annealed at 700 °C for 2 h under Ar protection, and then, the product and sulfur with a mass ratio of 1:2 were put into two separate sections of ceramic boat with sulfur at the upstream of the furnace. The furnace was heated to 500 °C for 2 h under Ar flow with a heating rate of 2 °C min^−1^. For comparison, pure MnS material was prepared using the same method, using commercial MnO_2_ as the raw material. The pure N, S-co-doped carbon (NSC) material was prepared using 2 M HCl to etch away the MnS in the MnS@NSC composite. The control samples with different MnS contents were also prepared following the same procedures except with varied refluxing time of 5 and 16 h; the resultant samples are defined as MnS@NSC-5 and MnS@NSC-16, respectively.

#### Preparation of NC Cathode Material

C_3_N_4_ was first obtained by annealing urea at the temperature of 550 °C for 2 h under Ar flow. Then, 0.25 g C_3_N_4_ was mixed with 50 mL of distilled water and sonicated for 30 min, followed by adding 2 g of wheat flour and 2 g KOH. The prepared mixture was stirred for 30 min and then dried at 80 °C. The resultant dry gel was carbonized at 800 °C for 1, 2, and 3 h under argon atmosphere, which was defined as NC-800-1, NC-800-2, and NC-800-3, respectively. After cooling down, the obtained NC material was washed with 2 M HCl and distilled water, respectively, to remove the impurity and then dried at 80 °C under vacuum for 10 h. For comparison, another NC was prepared by increasing the mass of KOH to 2.5 g with the annealing time of 2 h, which was defined as NC-800-2-2.5.

### Materials Characterization

The morphology and nanostructures of the samples were recorded by field emission scanning electron microscope (FESEM, JEOL 7600F) and transmission electron microscope (TEM, JEOL 2010 and JEM-2100F). Raman spectroscopy was conducted on a confocal Raman spectrometer with an excitation wavelength of 633 nm. The detailed crystal structures of the materials were measured by a Shimadzu powder diffractometer with Cu *Ka* radiation (*λ = *1.5406 Å). The X-ray photoelectron spectroscopy (XPS) analysis was carried out on a VGESCALab220i-XL spectrometer with a monochromatic Al *Ka* radiation (*hv *= 1486.7 eV). For the ex situ XPS, oxygen plasma etching was conducted to remove any possible SEI layer or oxidation layer on the samples. The nitrogen sorption isotherms and Brunauer–Emmett–Teller (BET) surface area were measured using a Tristar II 3020 instrument at liquid nitrogen temperature. Thermogravimetric analysis (TGA) was performed in air atmosphere from room temperature to 600 °C with a rate of 10 °C min^−1^ using a TA Q500.

### Electrochemical Measurements

The working electrodes were prepared by mixing 75 wt% active material, 15 wt% carbon black, and 10 wt% polyvinylidene fluoride (PVDF) in N-methyl pyrrolidinone (NMP) to form a slurry. The slurry was later casted on current collectors and then dried in vacuum oven at 80 °C for 12 h. The mass loading of the active materials was about 1 mg cm^−2^. The electrochemical properties were carried out in a CR2032-type coin cells which were assembled in an Ar-filled glove box. Pure sodium metal foil was used as both counter and reference electrodes; a glass microfiber filter was used as the separator, and 1 M NaClO_4_ in ethylene carbonate and diethyl carbonate (EC/DMC, 1:1, v:v) with 5 wt% fluoroethylene carbonate (FEC) as electrolyte. CV curves and EIS were recorded on electrochemical workstation (Autolab PGSTAT30). The galvanostatic charge/discharge and cycle life were performed on Neware battery-testing system. Galvanostatic intermittent titration technique (GITT) was performed by discharging the cells for 1200 s at 0.1 A g^−1^, followed by 1800 s relaxation. The SIC full cells were also fabricated into coin cells using a pre-sodiated MnS@NSC electrode (for 5 cycles at 0.1 A g^−1^ in a half-cell and then discharged to 0.01 V vs. Na/Na^+^) as anode, NC as the cathode, and the same separator/electrolyte as half-cell. The energy density and power density of the SICs were calculated based on the total mass of active materials in both anode and cathode. All the electrochemical measurements were tested at room temperature. The voltage of the LEDs is 1.9 V, and 10 LEDs were powered by our SICs for 7 min.

## Results and Discussion

**Fig. 1:**
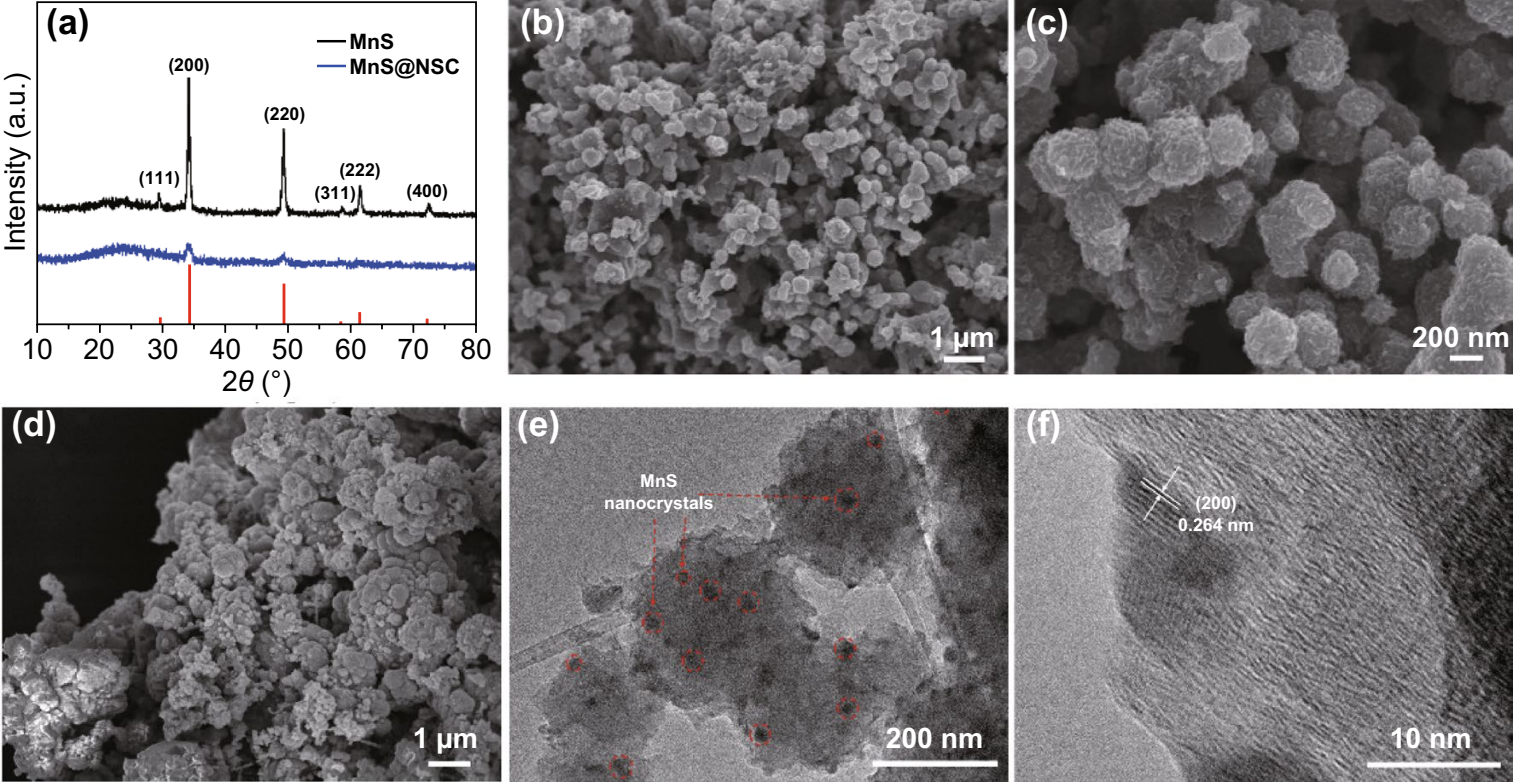
**a** XRD patterns of MnS and MnS@NSC. **b**, **c** SEM images of MnS@NSC. **d** SEM image of MnS. **e** TEM and **f** HRTEM images of MnS@NSC composite

The detailed preparation process of the intended MnS@NSC composite is elucidated in the Experimental section. The crystal composition of the samples was first analyzed by X-ray diffraction (XRD). All diffraction peaks can be ascribed to the cubic MnS phase (JCPDS No. 01-1089) without any impurities, indicating that a high purity crystalline phase of MnS has been synthesized, as shown in Fig. 1a [35, 38]. For the composite sample, the peaks located at degrees 34.3°, 49.2°, and 61.7° correspond to (200), (220), and (222) planes of MnS, respectively, indicating the presence of MnS in the composite. XRD patterns of MnS@NSC-5 and MnS@NSC-16 were also tested, as shown in Fig. S3. Considering that the broadening of the diffraction peaks is related to the size reduction of crystallites [35], the XRD result indicates that the embedded MnS nanoparticles are of small crystallite sizes compared to the prepared pure MnS sample. The morphology and nanostructures of the MnS@NSC and MnS were investigated using FESEM and TEM, as displayed in Fig. 1b–f. It can be clearly seen that the MnS@NSC is exclusively made of interconnected nanosize particles (Fig. 1b, c). The nanoparticles have rough surface, which can expose more active sites for sodium storage, as displayed in high-magnification SEM image shown in Fig. 1c. For the pure MnS sample shown in Fig. 1d, bigger and random nanoparticles of a few micrometers were observed. Figure 1f presents the high-resolution TEM (HRTEM) image of MnS@NSC; it can be clearly identified that MnS nanocrystals have been embedded into the amorphous carbon matrix (NSC), with the *d*-spacing of 0.264 nm corresponding to (200) planes of MnS. The scanning transmission electron microscopy (STEM) image and energy-dispersive X-ray spectroscopy (EDX) mapping (Fig. S1) of MnS@NSC also confirmed that the MnS nanocrystals were uniformly distributed in the NSC matrix. As shown in Figs. S2 and 1e, the TEM images further prove that the primary nanosized particles are interconnected and the MnS nanocrystals are fully encapsulated into the NSC matrix. The interconnected structure can benefit electron transport and Na^+^ diffusion, thus boosting the rate performance. The small crystallite size of MnS is anticipated to provide high electrochemical activity and high utilization of electrode materials, favoring fast Na^+^ diffusion and releasing local strain. The morphology and nanostructures of the MnS@NSC-5 and MnS@NSC-16 were also investigated using SEM and are displayed in Fig. S3. After etching and dissolving the MnS nanocrystals from the nanocomposite sample, the residue NSC material remains the same morphology and no diffraction peaks of MnS can be detected using XRD, as displayed in Fig. S4a, b. The content of electrochemically active MnS in MnS@NSC composites was quantified with TGA in air atmosphere. As demonstrated in Fig. S5, the initial weight loss of MnS@NSC at about 25–150 °C is ascribed to the evaporation of surface adsorbed water [35, 44]. The subsequent weight loss at approximately 200–550 °C should be due to the combustion of carbon and transformation of MnS to Mn_2_O_3_ in the MnS@NSC composite [35, 45]. Transformation of MnS to Mn_2_O_3_ was verified by the XRD pattern (Fig. S6) of sample MnS@NSC after TGA test. Thus, the content of MnS in MnS@NSC-5, MnS@NSC, and MnS@NSC-16 can be calculated as 14.8, 39.5, and 51.5 wt%, respectively.

**Fig. 2:**
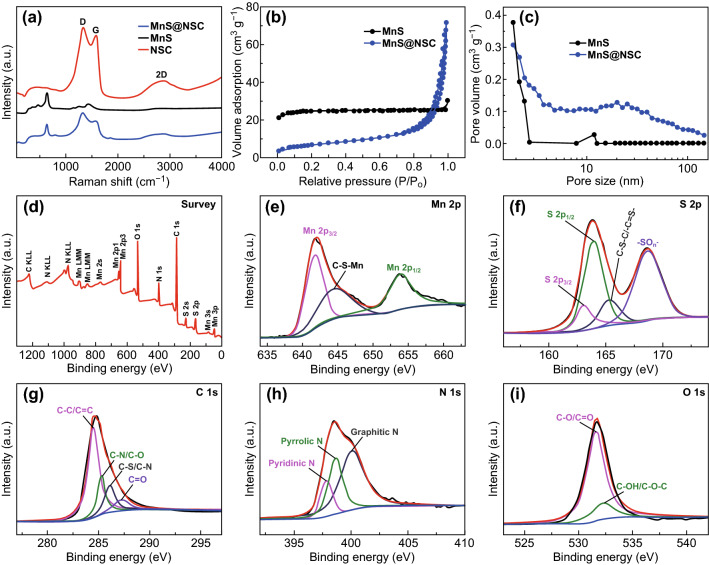
**a** Raman spectra of NSC, MnS, and MnS@NSC. **b** N_2_ adsorption/desorption isothermal curves and **c** pore size distribution profiles of MnS and MnS@NSC. **d** Typical XPS survey spectrum and corresponding **e** Mn 2*p*, **f** S 2*p*, **g** C 1*s*, **h** N 1*s*, and **i** O 1*s* spectra of MnS@NSC composite

Raman spectroscopy is further employed to detect the existence of MnS and carbon in the samples, as displayed in Fig. 2a. A characteristic peak at 633 cm^−1^ can be observed, which is assigned to intrinsic vibration modes of MnS that are present in both pure MnS and MnS@NSC samples [45, 46]. The peaks around 1338.7 and 1583.2 cm^−1^ in MnS@NSC correspond to the D band of disordered carbon and G band of graphitized carbon), respectively [6, 46]. This result is consistent with the NSC sample. It is widely accepted that D band is in association with amorphous carbon, where symmetry and selection are broken by defects or heteroatom doping, and G band is ascribed to the *E*_2g_ vibrations of graphite [13, 43]. The strong intensity of D band indicated that the heteroatom doping in NSC and MnS@NSC leads to the low crystallinity of carbon. The presence of 2D peaks in MnS@NSC and NSC is possibly related to the graphitization in the NSC materials. The surface area and pore structures of the samples were measured using the nitrogen adsorption/desorption technique, as demonstrated in Fig. 2b, c. The BET surface areas of MnS and MnS@NSC are 75.7 and 38.4 m^2^ g^−1^, respectively. The small surface area of MnS@NSC sample is tentatively ascribed to the formation of dense carbon nanospheres matrix. The MnS sample has more mesopores with pore sizes below 3 nm, while the MnS@NSC composite shows a hierarchical porous structure with the coexistence of micropores, mesopores, and macropores with pore size ranging from 1 to 90 nm, as shown in Fig. 2c. The multi-level porous structure of MnS@NSC is believed to be conducive to electrolyte penetration and favorable for the ionic diffusion, buffering the volumetric variations during the sodiation/desodiation processes, hence promoting the Na-storage kinetics and properties. In addition, the surface chemical composition and elemental valence states of MnS@NSC were investigated using XPS. The survey spectrum confirms the presence of Mn, S, C, N, and O elements, as displayed in Fig. 2d. The high-resolution spectrum of Mn 2*p* level (Fig. 2e) can be resolved into three peaks located at 653.9, 644.4, and 641.6 eV. The two bands at 653.9 and 641.6 eV correspond to Mn 2*p*_1/2_ and Mn 2*p*_3/2_, respectively, demonstrating the presence of Mn^2+^ [35, 45]. The small peak displayed at 643.8 eV may represent the C-S-Mn bond between MnS and carbon [45, 46]. As for S 2*p* core-level spectrum (Fig. 2f), the three obvious peaks centered at 163.1, 163.9, and 165.3 eV are assigned to S 2*p*_3/2_, S 2*p*_1/2_, and C–S–C/–C=S–, respectively, revealing the formation of MnS and the presence of sulfur covalently bonded to carbon with a heterocyclic configuration [45–47]. The peak around 168.6 eV can be indexed to the oxidized sulfur species (–SO_n_–), indicating the presence of oxygen in MnS@NSC [40, 45]. In the C spectrum (Fig. 2g), the peaks located at 284.5, 285.3, 286.1, and 287.3 eV are ascribed to C–C/C=C, C–N/C–O, C–S/C–N, and C=O, respectively [35, 43, 48]. The presence of C–N and C–S bond indicates that nitrogen and sulfur are successfully doped into carbon structure, which are expected to improve the electronic conductivity in NSC. As shown in Fig. 2h, the N 1*s* spectrum can be resolved into three peaks centered at 397.8, 398.6, and 400.2 eV, presenting pyridinic-, pyrrolic-, and graphitic-type N atoms, respectively, which further proved that nitrogen is successfully doped into carbon [43, 44]. The high-resolution O 1*s* spectrum revealed the presence of both C–O/C=O (531.6 eV) and C–OH/C–O–C (532.2 eV) bonds (Fig. 2i) [43, 45]. The presence of these bonds demonstrates the existence of low oxygen coordination defect sites in the MnS@NSC. The elemental analysis demonstrates that the N, S, and O contents in NSC are 4.8%, 3.2%, and 7.1%, respectively (Fig. S7). Heteroatom doping can generate more external defects and active sites, increasing the reactivity and electronic conductivity of carbon material, thus improving both sodium storage capacities and rate capability [13, 20, 45]. N, S co-doping can enhance the electrical conductivity of carbon materials. The O doping serves to generate more defects and active sites for adsorbing Na^+^, thus enhancing the sodium storage capacities. The NSC matrix introduces active sites for the growth of MnS nanocrystals, increases the charge-carrier concentration due to defects generation, and improves the electronic conductivity, making it favorable to boost the electrochemical performance of MnS@NSC composite.

**Fig. 3:**
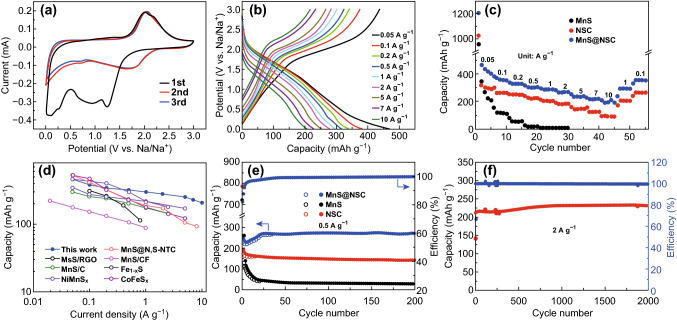
**a** First three cycles of MnS@NSC electrode at the scan rate of 0.2 mV s^−1^. **b** Charge/discharge curves of MnS@NSC electrode at different current densities. **c** Rate performance of NSC, MnS, and MnS@NSC. **d** Electrochemical performance of MnS@NSC comparison with literature reported metal sulfides. **e** Cycling performance of NSC, MnS, and MnS@NSC electrodes at current density of 0.5 A g^−1^. **f** Long-term cycling stability test of MnS@NSC at current of 2 A g^−1^

In order to investigate the sodium storage properties, the as-prepared MnS@NSC and control samples (pure MnS and NSC) were first evaluated in SIB half-cells. The first three cyclic voltammetry (CV) scans of MnS@NSC composite within 0.01–3 V at scan rate of 0.2 mV s^−1^ were performed, as shown in Fig. 3a. In the first cycle, one obvious broad peak at potential lower than 1.5 V was noted, which can be assigned to the insertion of Na^+^ into the lattice of MnS and the solid–electrolyte interface (SEI) layer formed on the surface of electrode materials [35, 43]. This peak disappeared in subsequent cycles, demonstrating the formation of stable SEI layers. The redox peaks located at 1.78/2.04 V could be ascribed to the reversible sodiation/desodiation in MnS [35, 45]. The second and third CV curves are well overlapped, demonstrating the highly reversible electrochemical reaction and remarkable cycling stability of MnS@NSC electrode. As displayed in Fig. S8, the CV curves of NSC are also tested for comparison. The redox peaks located at 1.45/1.97 V should be attributed to the reversible conversion reaction of Na with S doped in NSC to one or to several Na-S compounds, which is well consistent with previously reported S-doped carbon in Na-storage and Na-S battery [13, 36, 49]. The different electrochemical behavior and notably higher current values of MnS@NSC than NSC demonstrate that the improved electrochemical activity of MnS@NSC is contributed to MnS (MnS + 2Na ↔ Na_2_S + Mn). The first five galvanostatic charge/discharge (GCD) profiles of MnS@NSC are displayed in Fig. S9. The capacity loss in the first cycle may be ascribed to the electrolyte decomposition, SEI formation, and irreversible phase conversion during the Na^+^ insertion process [13, 22]. This issue can be avoided using presodiation technique before assembling full SIBs and hybrid devices. The typical GCD profiles versus current densities of MnS@NSC are demonstrated in Fig. 3b. All the curves display a charge/discharge plateau, which is in agreement with the CV results in Fig. 3a. The typical GCD profiles of NSC and MnS are also tested and displayed in Figs. S10 and S11. The rate capability of MnS@NSC, NSC, and MnS is compared and shown in Fig. 3d. The as-prepared MnS@NSC can demonstrate a high capacity of 464.3 mAh g^−1^ at 0.05 A g^−1^; the capacity can still retain 205.6 mAh g^−1^ at 10 A g^−1^, corresponding to a capacity retention of 44.3%, demonstrating its excellent rate performance. The rate performance of MnS@NSC-5 and MnS@NSC-16 was also tested as shown in Fig. S12, indicating the optimum electrochemical performance of MnS@NSC. The capacity of the composite samples was calculated based on the total weight of composite, including both MnS and NSC. On the other hand, the rate performance of NSC electrode is inferior (317.2 mAh g^−1^ at 0.05 A g^−1^ and 93.2 mAh g^−1^ at 10 A g^−1^). For the pure MnS, due to the large polarization, the capacity is decreased rapidly with the increased current density. When compared with the previously reported MnS-based sodium-ion anode materials (Fig. 3d), such as MnS@N, S-co-doped carbon nanotube [35], MnS/RGO [42], MnS/CF [38], and MnS/C [41], the present MnS@NSC anode displays apparent superiority in both capacity and rate capability. This value is also comparable or better than some other metal sulfides, for example, Fe_1-x_S [16], NiMnS_x_ [9], and CoFeS_x_@C [8] (Fig. 3d). The cycling stability of the as-prepared MnS@NSC, NSC, and MnS electrodes at 0.5 A g^−1^ was performed and shown in Fig. 3e. Both MnS@NSC and NSC exhibit good cycling stability, and reversible capacity of 269.5 and 144.6 mAh g^−1^ is maintained for MnS@NSC and NSC after 200 cycles, respectively. As for the capacity of MnS@NSC, it is increased in the first several tens of cycles and this could be related to the activation process caused by gradual electrolyte penetration and the increased active surface induced by nanosize effects that have been reported in previous works [8, 9, 35]. However, for the MnS electrode, the capacity is soon reduced to less than 28.3 mAh g^−1^ after 200 cycles. The poor cycling stability can be ascribed to the large volume changes and provoked side reactions of carbonate-based electrolyte with anions in the charge/discharge process, in agreement with the previous studies [8, 16, 37]. For MnS@NSC, the carbon matrix not only can enhance the electrical conductivity and buffer the volume changes, but also can inhibit side reactions by limiting the dissolution of polysulfides. The presence of carbon matrix with micro-/mesopores can physically adsorb as-formed polysulfides and act as a physical barrier to inhibit the dissolution of polysulfides into electrolyte, thus delivering excellent rate performance and long-term stability. Figure 3f depicts the high-rate cycling test of MnS@NSC at 2 A g^−1^. The MnS@NSC electrode displays excellent cycling stability and reversibility without obvious capacity decay after 2000 cycles. As shown in Fig. S13, nanosphere morphology of MnS@NSC was still maintained after 1000 cycles, indicating that NSC can maintain the structural integrity.

**Fig. 4:**
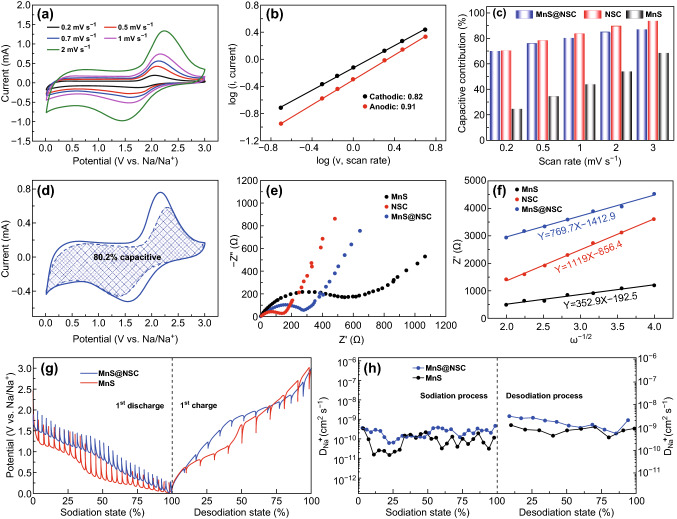
**a** CV curves of MnS@NSC electrode at different scan rates. **b** Analysis of *b* value for cathodic and anodic peaks of MnS@NSC. **c** Capacitive contribution of MnS@NSC, NSC, and MnS electrodes at different scan rates. **d** Capacitive contribution of MnS@NSC electrode at 1 mV s^−1^. **e** Nyquist plots of NSC, MnS, and MnS@NSC electrodes. **f** The relationship plots of *Z*′ versus *ω*^−1/2^ at low-frequency region for NSC, MnS, and MnS@NSC electrodes. **g** GITT curves (repeated discharging the cells for 1200 s at 0.1 A g^−1^ followed by 1800 s relaxation) and **h** corresponding Na^+^ diffusion coefficient at different discharge/charge states of MnS and MnS@NSC electrodes

Based on the above results, to investigate the reaction kinetics of MnS@NSC, MnS, and NSC electrodes, the CV curves versus scan rates were recorded (Figs. 4a and S14–S16), respectively. As shown in Figs. 4a and S14, both the redox peak current and potential polarization of MnS@NSC electrode are increased with scan rates raised from 0.2 to 30 mV s^−1^. The redox capacitive contribution can be analyzed from Eqs. 1 and 2 [4, 16, 50–53]:

1 $$i\left( v \right) = av^{b}$$

2 $$i\left( v \right) = k_{1} v + k_{2} v^{1/2}$$

where *a* and *b* are constants, *i*(*V*) and *v* are the current and the scan rate (mV s^−1^), and *k*_1_ and *k*_2_ are the fitting values. A *b* value close to 0.5 demonstrates that the charge storage is contributed by solid-state diffusion-controlled process, while the *b* value equal to 1 signifies a capacitive-controlled behavior. As shown in Fig. 4b, the *b* values of cathodic peak and anodic peak of MnS@NSC electrode are calculated as 0.82 and 0.91 using Eq. 1, indicating that the capacitive Na^+^ storage dominates most of the charge storage. To be more accurate, the overall contributions from capacitive processes versus scan rates can be calculated using Eq. 2. As displayed in Fig. 4c, the capacitive contributions in MnS@NSC electrode are 69.9%, 75.9%, 80.2%, 84.7%, and 86.8% at scan rates of 0.2, 0.5, 1, 2, and 3 mV s^−1^, respectively. These values are higher than the pure MnS electrode and lower than the NSC electrode (Fig. 4c), indicating that downsizing of MnS can offer abundant active sites and NSC encapsulation can improve the electrical conductivity of MnS for fast capacitive sodium storage, thus boosting the rate performance. With increased scan rates, the capacitive contribution is increased as expected, illustrating that the capacitive sodium-ion storage kinetic occupies a large proportion at high scan rates. For instance, Figs. 4d and S17 illustrate the typical voltage profiles for the capacitive regions of MnS@NSC and NSC at scan rate of 1 mV s^−1^, revealing that 80.2% and 83.7% of the total capacity stems from the capacitive behavior, respectively.

To further understand the kinetics of MnS@NSC composite, the electrochemical impedance spectroscopy (EIS) of MnS@NSC, MnS, and NSC was performed after three cycles; the Nyquist plots are illustrated in Fig. 4e. All the EIS curves have the similar shapes with a semi-circle in high-frequency region and a straight line in low-frequency region. Obviously, the MnS@NSC composite has a smaller charge-transfer resistance than pure MnS, demonstrating that the electron/ion transport is enhanced through downsizing of MnS and NSC encapsulation. Meanwhile, the sodium-ions diffusion coefficient can be investigated according to the Warburg factor (σ) related to the slope of the linear fittings at low-frequency region in Eq. 3 [36, 43],

3 $$Z^{\prime} = R_{e} + R_{c} + \sigma_{w} \omega^{ - 1/2}$$

As shown in Fig. 4f, the *Z*′–*w*^−1/2^ curves demonstrate that NSC and MnS electrodes have the maximum (1119) and minimum (352.9) slope values, while the MnS@NSC has a moderate (769.7) slope, indicating that MnS@NSC electrode has higher sodium-ion diffusion coefficient than MnS electrode. The enhanced sodium-ion transport kinetics can be ascribed to downsizing of MnS and encapsulation of NSC, which can shorten the ion diffusion pathway and promote the facile access of Na^+^/e^−^. The GITT was also carried out to evaluate the sodiation/desodiation ion diffusion kinetics, as shown in Fig. 4g, h. The results indicate that generally the sodium-ion diffusion coefficients during the whole sodiation/desodiation process in MnS@NSC are higher than those of pure MnS, which reveals the greatly enhanced sodium-ion diffusion kinetics in MnS@NSC, benefiting from the reduced size of MnS and improved electronic conductivity by NSC encapsulation. It is noticed that at some points, the sodium-ion diffusion coefficients of MnS@NSC are lower than those of MnS in the discharge process, which is tentatively ascribed to the Na^+^ insertion into bulk MnS that induced size reduction and more active sites exposure. This sodium-ion diffusion coefficients of MnS@NSC and bulk MnS in the second discharge/charge cycles are also quantified as shown in Fig. S18. The sodium-ion diffusion coefficients of MnS@NSC in the second cycle are higher than those of the pure MnS.

In order to study the electrochemical mechanism, the Mn 2*p* spectra of bulk MnS at different charge/discharge states in the first and second cycles were collected by ex situ XPS, as shown in Fig. S19. During the first discharge process to 0.01 V, an obvious downshift of the binding energies can be clearly seen. The Mn 2*p*_3/2_ peaks located at 641.6 eV of the pristine MnS have downshifted to 640.7 eV in the first discharge to 0.01 V, revealing the reduction of manganese oxidation state from 2+. A new peak located at 642.6 eV was deconvoluted upon discharge to 0.75 V and was absent after further discharge to 0.01 V, demonstrating the formation of intermediate Na_x_MnS (where x < 1). Ex situ XRD and TEM were also conducted to reveal the electrochemical mechanism of MnS. As shown in Fig. S20a–c, the presence of Na_2_S and Mn can be clearly detected in the sample of MnS discharged to 0.01 V. The above result clearly shows that MnS undergoes combined intercalation and conversion process during discharge. When being recharged back to 3 V, the deconvoluted peaks in Mn spectra restored to higher binding energies, indicating the increased oxidation states of Mn that is possibly correlated with reformation of MnS after desodiation. After discharge to 0.01 V in the second cycle, the binding energy of Mn was again reduced, repeating the discharge process in the first cycle. This result is in good agreement with the CV curves shown in Fig. S15a; the first two cathodic peaks are related to the Na^+^ intercalation reaction, leading to the formation of intermediate Na_x_MnS phase with reduced oxidation states. The third reduction peak is assigned to conversion reaction, with notably reduced binding energies.

In addition, N-doped 3D porous carbon (NC) cathode material was synthesized using C_3_N_4_ and biomass wheat flour as nitrogen and carbon source (Fig. S21), respectively. The annealing time and mass ratio of wheat flour to KOH were optimized (Figs. S22–S26, Table S1). The NC material with a carbonation time of 2 h and a wheat flour/KOH ratio of 1:1 demonstrated the highest performance. As displayed in Fig. S25a, the NC material can demonstrate a highest capacity of 108.8 mAh g^−1^ at 0.1 A g^−1^ and maintains 62.3 mAh g^−1^ at 10 A g^−1^, implying its outstanding rate performance and fast ion adsorption/desorption from the large surface area (2846 m^2^ g^−1^ in Fig. S24a, b). Furthermore, after 5000 cycles at current of 3 A g^−1^, the capacity of NC shows minor loss and coulombic efficiency retains nearly 100% (Fig. S25b), demonstrating the highly reversible adsorption/desorption process.

**Fig. 5:**
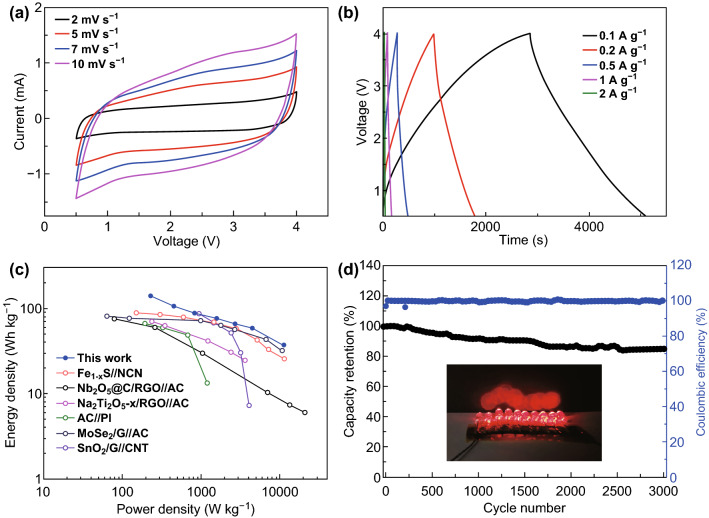
**a** CV curves of MnS@NSC//NC SIC from 2 to 10 mV s^−1^. **b** Charge/discharge profiles of MnS@NSC//NC SIC from 0.1 to 2 A g^−1^. **c** Ragone plots of the SIC versus literature reported SICs. **d** Cycling stability test of the SIC at current of 2 A g^−1^. The inset is the demonstration of 10 red LEDs powered by a charged SIC

The excellent electrochemical performance of MnS@NSC anode and NC cathode is harvested further in SIC. As a proof of concept, a full cell of SIC is fabricated to demonstrate its practical applications. Before assembling the SIC, the MnS@NSC electrode was pre-cycled for five cycles and then discharged to 0.01 V at 0.1 A g^−1^ in a Na half-cell to harness high efficiency. Considering the charge balance between anode and cathode, the mass ratio of the active materials in two electrodes is fixed to 1:3. A suitable voltage window of 0.5–4 V of the SIC is controlled to avoid the possible electrolyte decomposition and side reactions. The CV curves of the SIC ranging from 2 to 10 mV s^−1^ were measured and are displayed in Fig. 5a. The CV curves presented a slight deviation from the rectangular shape, unlike the conventional symmetric SC, demonstrating the combination of two different charge storage mechanisms of faradaic and non-faradaic reactions. As the scan rate increases, the shape of CV curves is still retained without serious distortion, suggesting its high reversibility and excellent rate capability. The charge/discharge profiles at various current densities display nearly symmetric quasi-triangular shapes as depicted in Fig. 5b, which again prove the combination of two different charge storage behaviors, agreeing well with the CV results. The energy and power densities of the SIC were calculated, and the corresponding Ragone plot (energy *vs.* power) is presented in Fig. 5c. As expected, the MnS@NSC//NC SIC device can demonstrate high energy density of 139.8 Wh kg^−1^ at power density of 230 W kg^−1^. The energy density can still maintain 36.4 Wh kg^−1^ at a high power density up to 11,500 W kg^−1^, demonstrating its superior rate performance. In comparison, our SIC also outperforms other previously reported SIC systems, such as Fe_1-x_S//NCN (87.8 Wh kg^−1^) [16], Nb_2_O_5_@C/RGO//AC (76 Wh kg^−1^) [54], Na_2_Ti_2_O_5-x_//RGO/AC (70 Wh kg^−1^) [23], AC//PI (66 Wh kg^−1^) [55], MoSe_2_/G//AC (82 Wh kg^−1^) [29], and SnO_2_/G//CNT (86 Wh kg^−1^) [21]. Furthermore, the SIC system also possesses an excellent durability with a capacity retention of 84.5% after 3000 cycles at 3 A g^−1^, with high coulombic efficiency of nearly 100% (Fig. 5d). As shown in Fig. 5d, 10 red light-emitting diodes (1.9 V) can be powered for 7 min by a single charged device, demonstrating the practical application of the hybrid system. The excellent electrochemical performance of the MnS@NSC//NC hybrid system can overcome the intrinsic limited kinetics and capacity mismatch between anode and cathode in SIC, demonstrating its great promise as energy storage device candidate with high power and energy density for future practical applications.

## Conclusions

In summary, we have demonstrated a high-rate SIB anode material by encapsulating MnS nanocrystals into the N, S-co-doped carbon matrix. The N, S-co-doped carbon can improve not only the conductivity of the hybrid material, but also can buffer the volume changes and agglomeration of MnS nanocrystals. The downsized MnS can expose more active sites for charge storage, enhance the diffusion kinetics, and promote pseudocapacitive behavior. Thus, the well-designed nanostructured MnS@NSC electrode displays a large specific capacity, excellent rate capability, and long-term stability as anode for SIB. A prototype SIC with MnS@NSC as anode and NC as cathode could deliver high energy and power densities (139.8 Wh kg^−1^ and 11,500 W kg^−1^, respectively), along with outstanding cycle life with 84.5% capacity maintaining after 3000 cycles. This study provides a new route to design high-performance metal sulfide-based electrode for application in high-power and energy density SICs.

## Electronic supplementary material

Below is the link to the electronic supplementary material.

Supplementary material 1 (PDF 1530 kb)
