# Supplementary material for: Encapsulation of MnS Nanocrystals into N, S-Co-doped Carbon as Anode Material for Full Cell Sodium-Ion Capacitors
Source: Nanomicro Lett. 2020 Jan 22;12:34. doi: 10.1007/s40820-020-0367-9 (PMC7770765; doi:10.1007/s40820-020-0367-9)
Supplement: Supplementary file 1 — Supplementary material 1 (PDF 1530 kb) [file 40820_2020_367_MOESM1_ESM.pdf]

Supporting Information for

Encapsulation of MnS Nanocrystals into N,S Co-doped Carbon as Anode Material for Full Cell Sodium-ion Capacitors

Shaohui Li[1, #], Jingwei Chen[1, 2, #], Jiaqing Xiong[1], Xuefei Gong[1], Jinghao Ciou[1], Pooi See Lee[1, 2, *]

[1]School of Materials Science and Engineering, Nanyang Technological University, Singapore 639798, Singapore

[2]Singapore-HUJ Alliance for Research and Enterprise (SHARE), Nanomaterials for Energy and Water Nexus (NEW), Campus for Research Excellence and Technological Enterprise (CREATE), 1 Create way, Singapore 138602, Singapore

[#]Shaohui Li and Jingwei Chen contributed equally to this work

*Corresponding author. E-mail: pslee@ntu.edu.sg (Pooi See Lee)

Supplementary Figures and Table

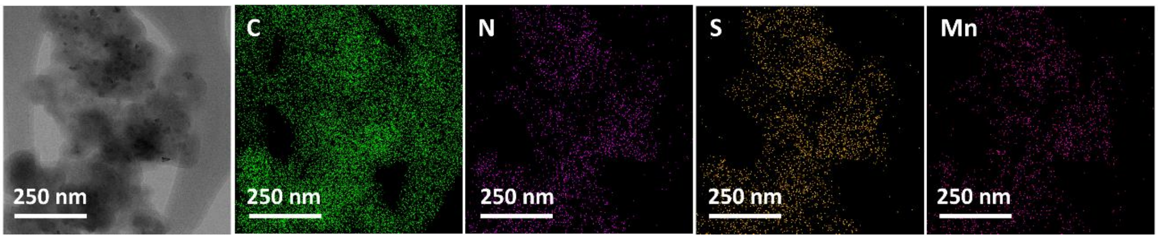

Fig. S1 STEM image and the corresponding EDX elemental mapping images of MnS@NSC

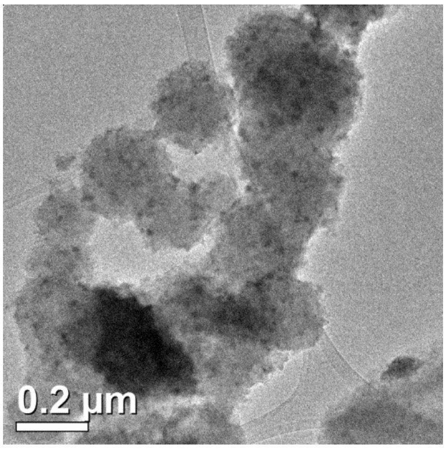

Fig. S2 TEM image of MnS@NSC

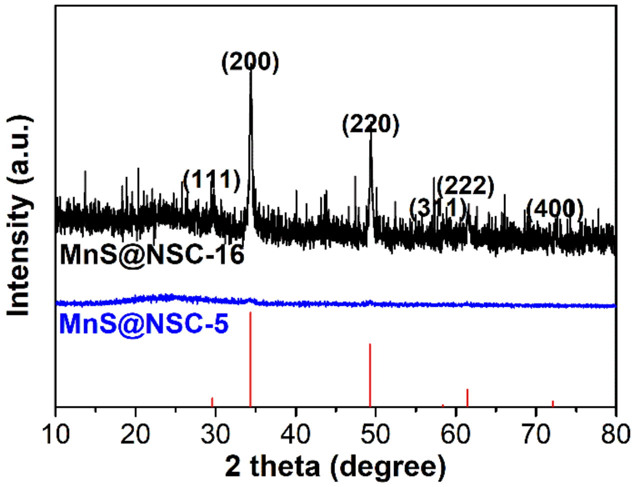

Fig. S3 XRD patterns of MnS@NSC-5 and MnS@NSC-16

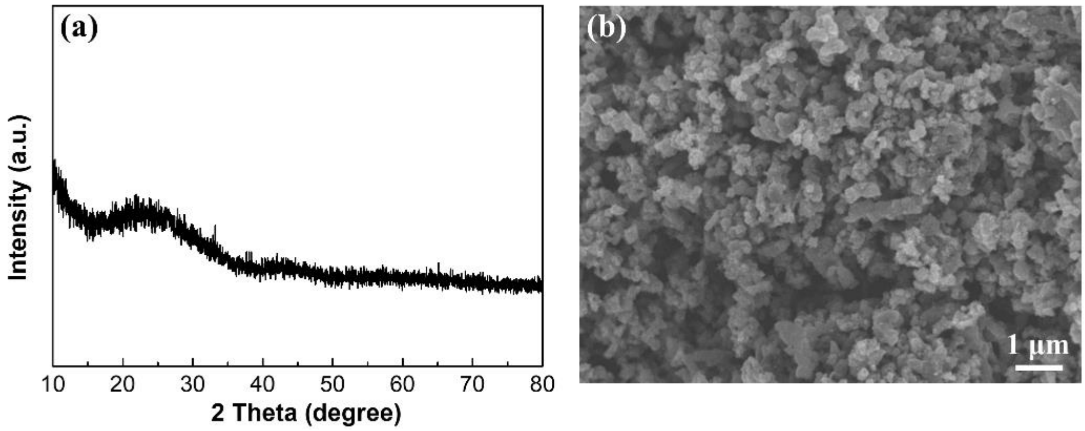

Fig. S4 a XRD and **b** SEM image of NSC

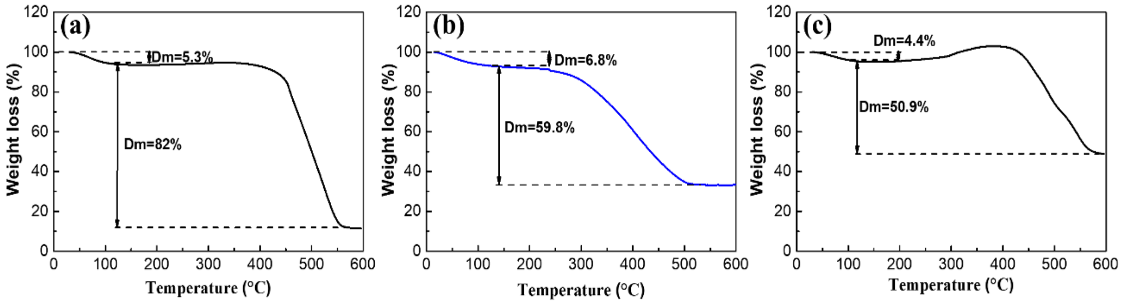

Fig. S5 TGA analysis of MnS@NSC-5 (**a**), MnS@NSC (**b**) and MnS@NSC-16 (**c**) composite materials in air flow

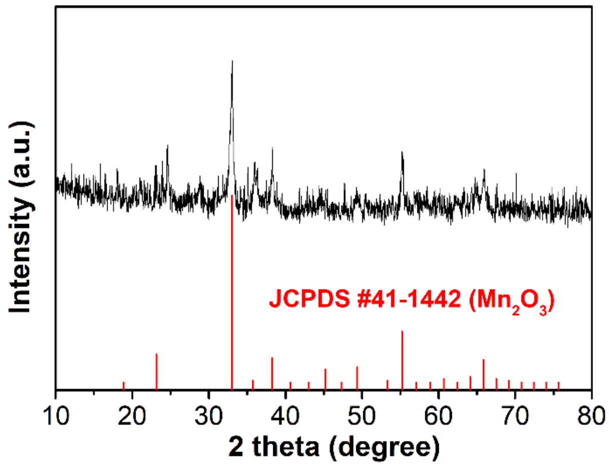

Fig. S6 XRD pattern of MnS@NSC after TGA analysis

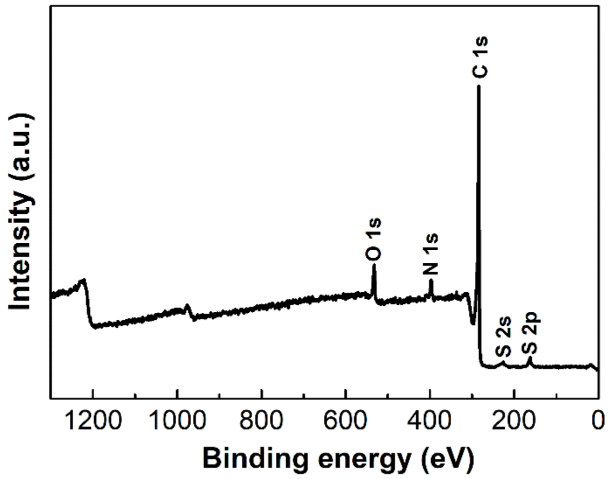

Fig. S7 Typical XPS survey spectrum of NSC material

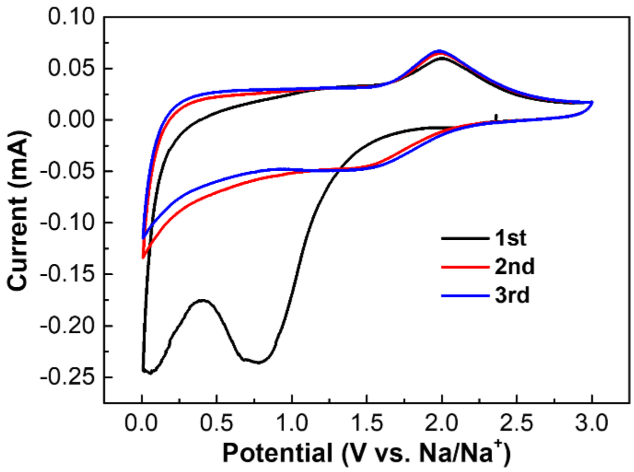

Fig. S8 First three CV curves of NSC at the scan rate of 0.2 mV s^{-1}

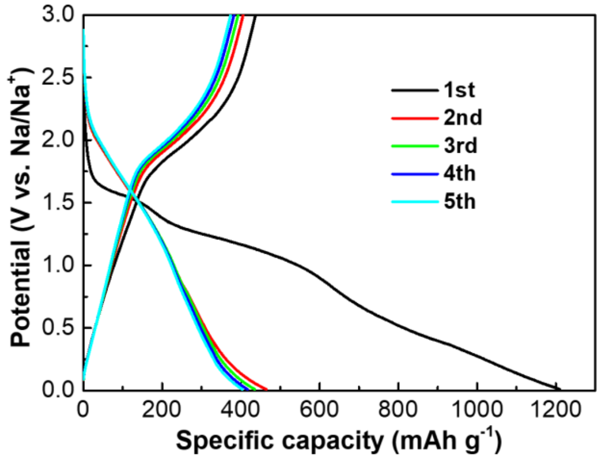

Fig. S9 First five charge/discharge profiles of MnS@NSC electrode

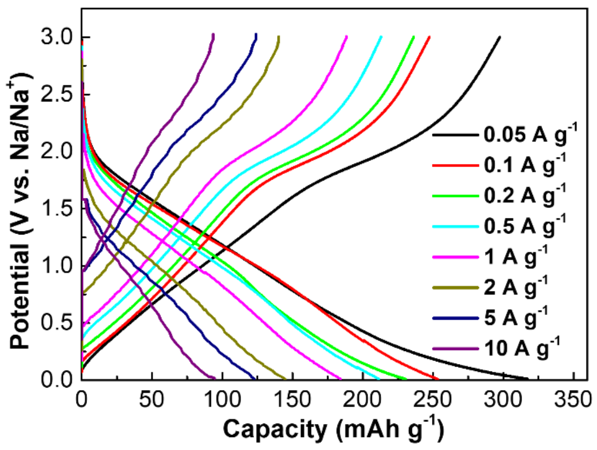

Fig. S10 Galvanostatic charge/discharge profiles of NSC with different current densities

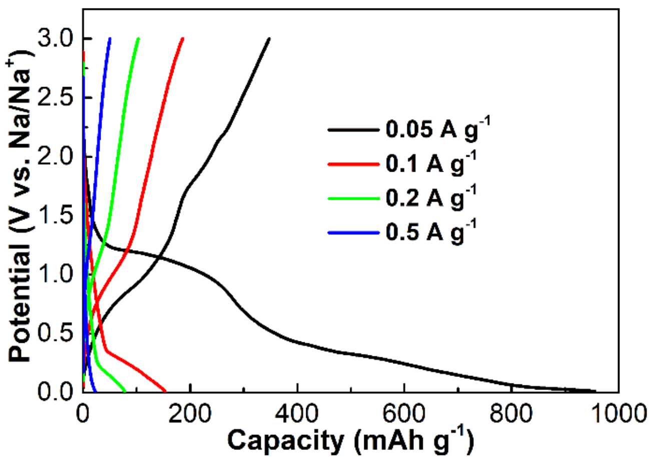

Fig. S11 Galvanostatic charge/discharge profiles of MnS with different current densities

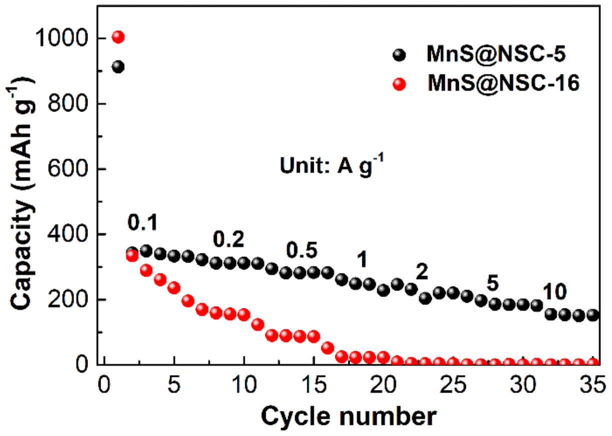

Fig. S12 Rate performance of MnS@NSC-5 and MnS@NSC-16

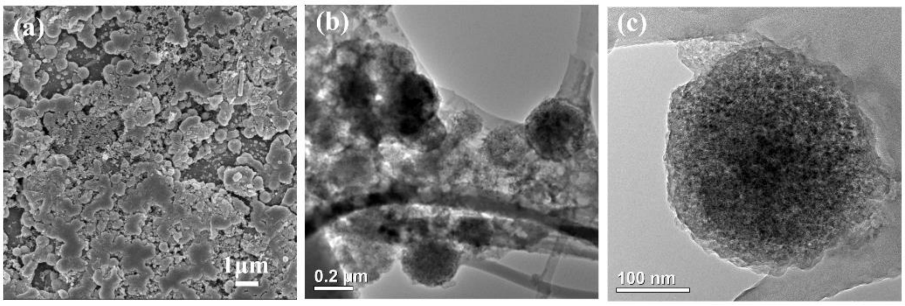

Fig. S13 SEM (**a**) and TEM (**b**) of MnS@NSC after 1000 cycles

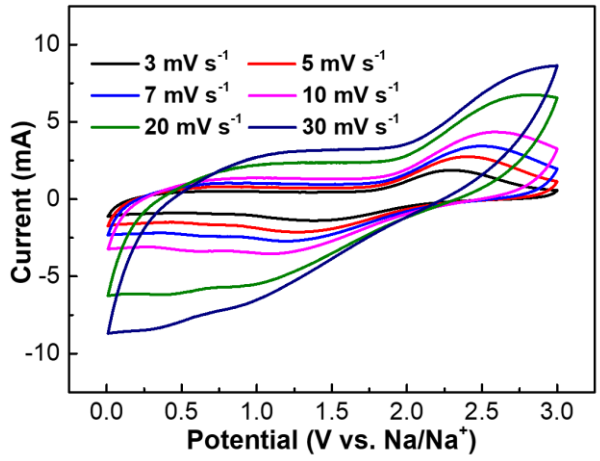

Fig. S14 CV curves of MnS@NSC electrode from 3 to 30 mV s^{-1}

The anodic and cathodic peaks shift significantly as the scan rate increases, which is ascribed to the diffusion-controlled contributions.

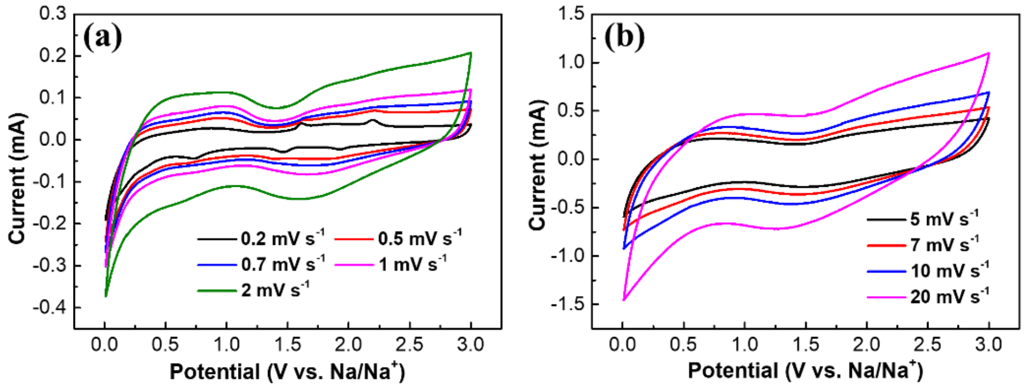

Fig. S15 a and **b** CV curves of MnS electrode at different scan rates

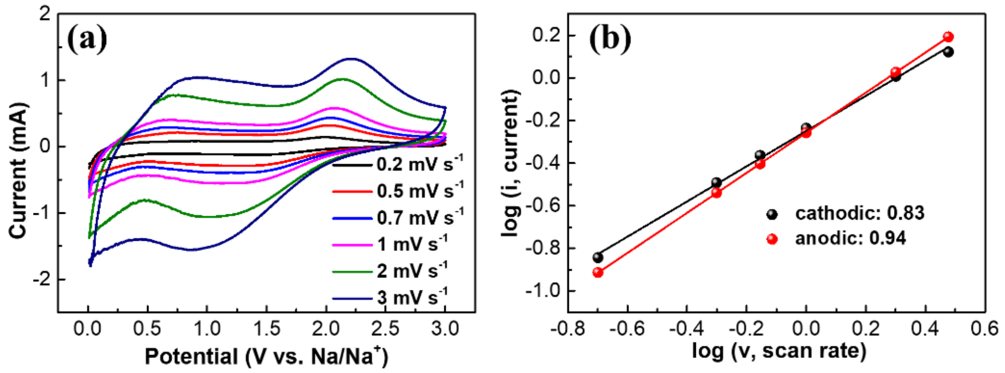

Fig. S16 a CV cures of NSC from 0.2 to 3 mV s^{-1}. **b** Analysis of b value for cathodic and anodic peaks of NSC

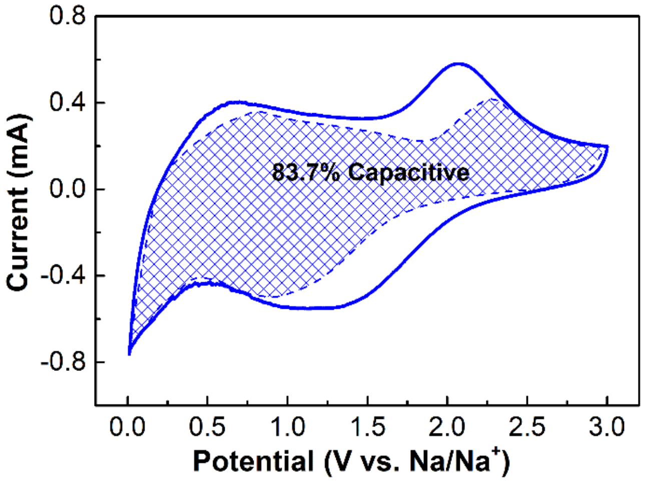

Fig. S17 Capacitive contribution of NSC electrode at 1 mV s^{-1}

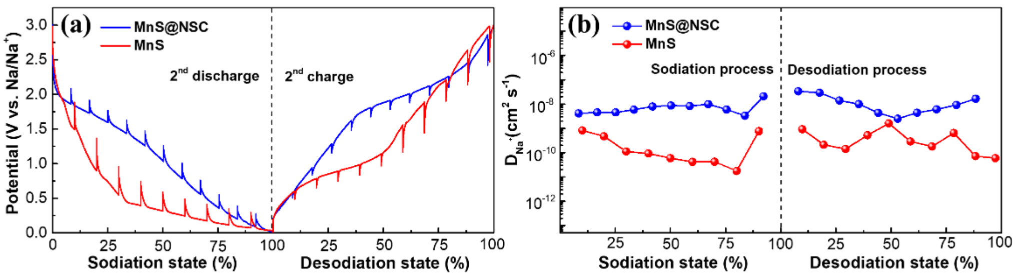

Fig. S18 a GITT curves and **b** corresponding Na^+ diffusion coefficient at different discharge/charge state of MnS and MnS@NSC electrodes in the second cycle

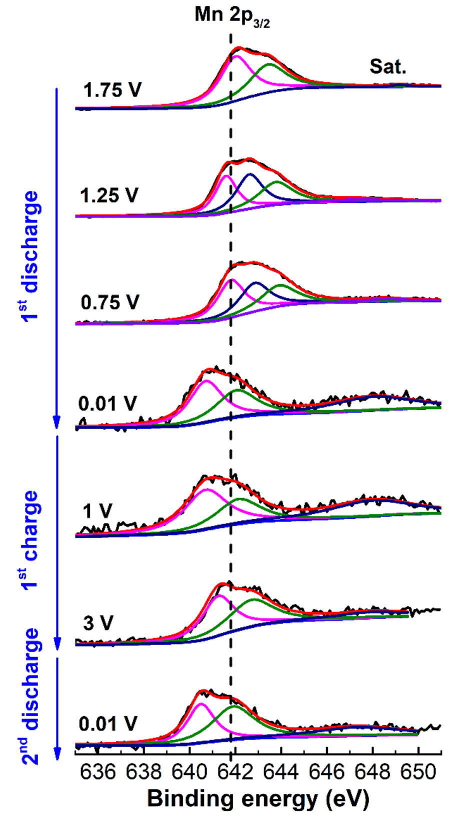

Fig. S19 Ex-situ XPS characterization of Mn spectra

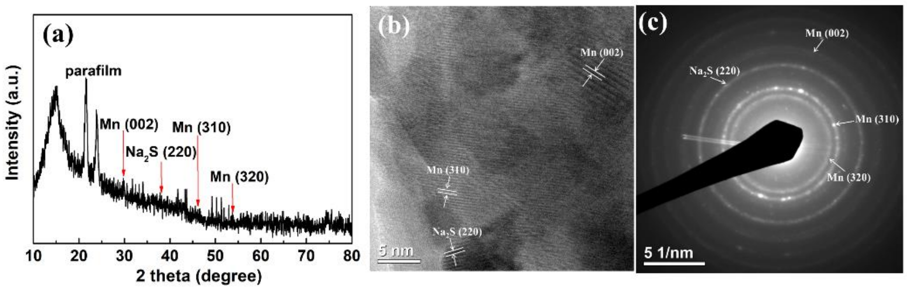

Fig. S20 XRD (**a**), HRTEM (**b**) and SAED (selected area electron diffraction) (**c**) of MnS@NSC electrode after discharged to 0.01 V

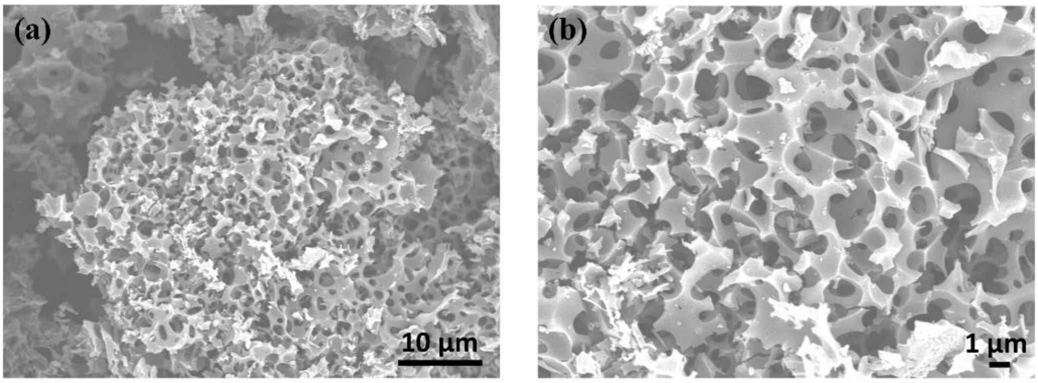

Fig. S21 a and **b** SEM images of NC material

As shown in Fig. S21 , the carbon material exhibits a 3D porous structure, which can expose more active sites for ion transport and storage, enabling fast charge and discharge, so as to boost the rate ability and capacity.

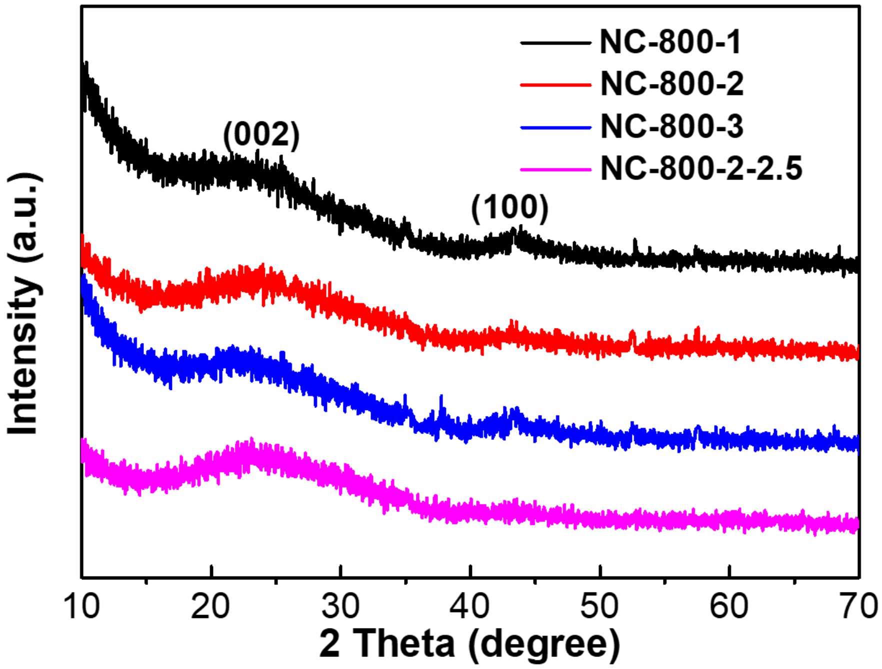

Fig. S22 XRD patterns of different NC materials

The XRD patterns of NC materials exhibits two broad diffraction peaks that are ascribed to the (002) and (100) planes of graphite. The notably broadened peaks demonstrated its low ordering.

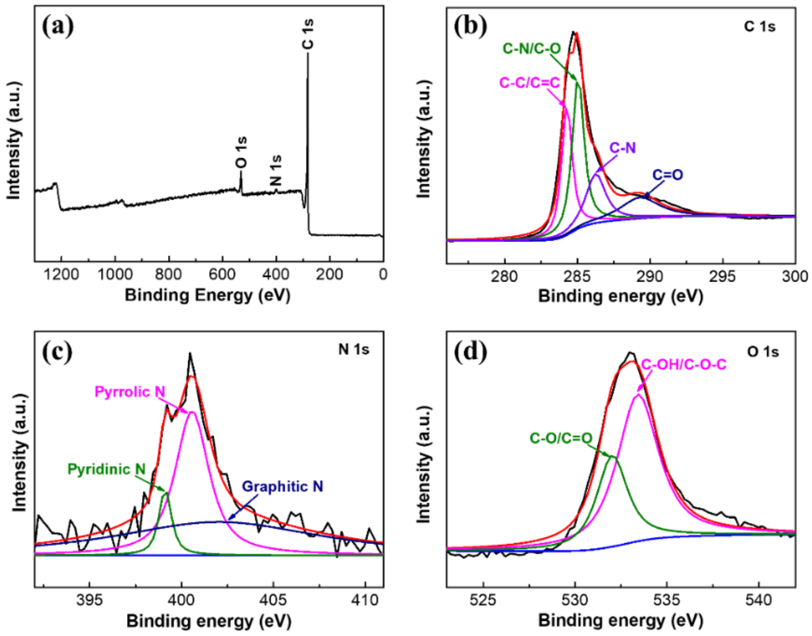

Fig. S23 a Typical XPS survey spectrum and corresponding **b** C 1s, **c** N 1s and **d** O 1s spectra of NC material

The full survey spectra of the as-prepared NC clearly demonstrates the presence of C, N, and O elements on the surface (Fig. S23a). The elemental analysis demonstrates that the N and O contents in NC are 6.8% and 1.4%, respectively. As shown in Fig. S23b, the C 1s peak can be resolved into four peaks located at 284.3, 285.1, 286.3, and 289.3 eV, corresponding to C-C/C=C, C-N/C-O, C-N and C-O, respectively [11, 37]. The N 1s peak (Fig. 23c) can be disintegrated into three peaks centered at 399.1, 400.6 and 402.1 eV, presenting pyridinic N, pyrrolic N and graphitic N, respectively, indicating that N was successfully doped into the carbon [37, 39]. According to XPS result, the total N content is about 1.8%. Moreover, in the O 1s spectrum (Fig. 23d), the peak can be decomposed into two peaks located at 532.1 and 533.4 eV, which are consistent with the binding energies of C-O/C=O and C-OH/C-O-C, respectively [37, 39].

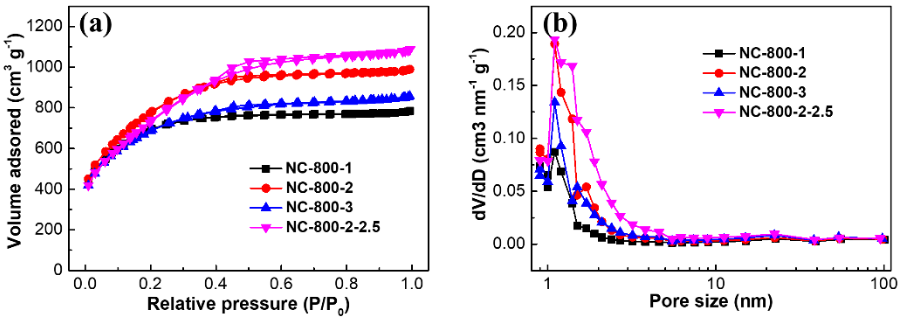

Fig. S24 a Nitrogen adsorption/desorption isotherms of NC materials. **b** Pore size distribution of NC materials

Table S1 The surface area of NC materials

	NC-800-1	NC-800-2	NC-800-3	NC-800-2-2.5
Surface area (m^2 g^{-1})	2494	2846	2479	2705

The nitrogen adsorption/desorption isotherms and pore size distribution of NC materials are shown in Fig. S24a, b, respectively. The detailed surface areas of the NC materias are listed in Table S1. As shown in Fig. S24a, all the four samples diplayed a type I isotherm curve, demonstrating its microporous structure [12, 30]. The surface area of the samples are firstly increased and then reduced when the annealing time was prolonged from 1 to 3 h. When the weight of KOH was increased to 2.5 g, the surface area is also reduced. The highest surface area of 2846 m^2 g^{-1} can be obtained for the sample of NC-800-2. Thus the optimized synthesis parameter of the KOH weight is 2 g and the annealing time is 2 h. The pore size of NC materials range from 1-5 nm (Fig. S24b), which is favorable for the ClO_4^- penetration and transportation, greatly improving the rate performance.

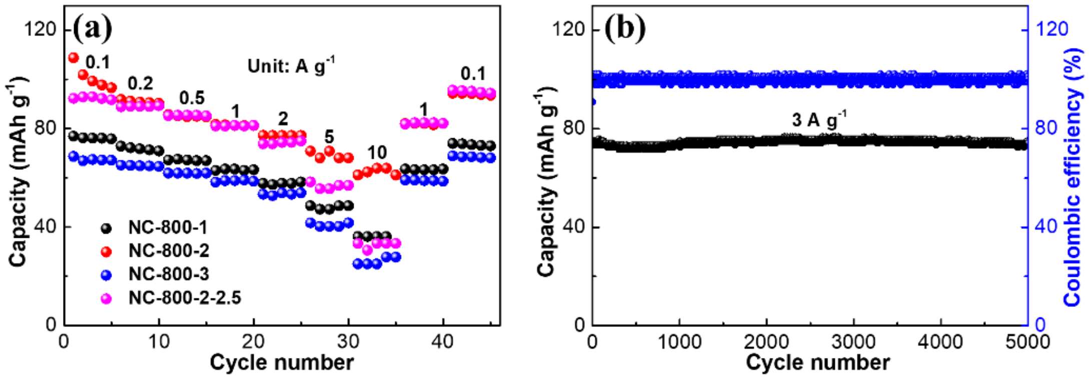

Fig. S25 a The rate performance of different NC cathode materials. **b** Cycling stability of NC-800-2 electrode at current density of 3 A g^{-1} for 5000 cycles

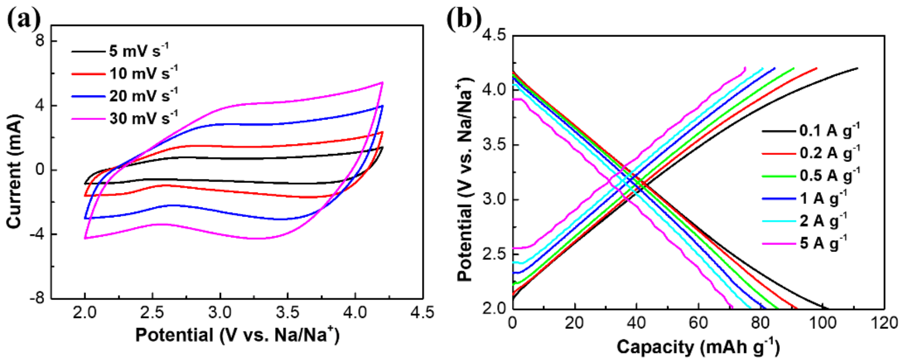

Fig. S26 a CV curves of NC-800-2 in the potential of 2.0-4.2 V at different scan rates. **b** Galvanostatic charge/discharge curves of NC-800-2 at different current density
